# Supplementary material for: Current Uses of Mushrooms in Cancer Treatment and Their Anticancer Mechanisms
Source: Int J Mol Sci. 2022 Sep 10;23(18):10502. doi: 10.3390/ijms231810502 (PMC9504980; doi:10.3390/ijms231810502)
Supplement: Supplementary file 1 [file ijms-23-10502-s001.zip › ijms-1869940-supplementary.pdf]

Supplementary Data—Table S1

Mushroom	Cancer	Phase	Study Status	Active Compound/s	Identifier	Investigator
Trametes versicolor	Breast cancer	Phase 4	Ongoing	Krestin, PSK, PSP	NCT00647075	Pere Gascon
Ganoderma lucidum	Breast cancer, quality of life	Phases 1 and 2	Ongoing	Polysaccharide peptides	NCT02486796	Christina Shannon
Grifola frondosa	Lung neoplasms	Phase 1	Ongoing	Grifolan, Maitake D Fraction	NCT02603016	Shunchang Jiao
Grifola frondosa	Breast carcinoma	Phase 2	Ongoing	Grifolan, Maitake D Fraction	NCT02603016	Shunchang Jiao
